# Optimizing restoration: A holistic spatial approach to deliver Nature’s Contributions to People with minimal tradeoffs and maximal equity

**DOI:** 10.1073/pnas.2402970121

**Published:** 2024-08-12

**Authors:** Trisha Gopalakrishna, Piero Visconti, Guy Lomax, Esther Boere, Yadvinder Malhi, Parth Sarathi Roy, Pawan K. Joshi, Giacomo Fedele, Ping Yowargana

**Affiliations:** ^a^Department of Geography, University of Exeter, Exeter EX4 4QE, United Kingdom; ^b^Biodiversity and Natural Resources Program, International Institute of Applied Systems Analyses, Laxenburg A-2361, Austria; ^c^Environmental Change Institute, School of Geography and the Environment, University of Oxford, Oxford OX1 3QY, United Kingdom; ^d^Global Systems Institute, University of Exeter, Exeter EX4 4QE, United Kingdom; ^e^Department of Environmental Geography, Instituut voor Milieuvraagstukken, Vrije Universiteit Amsterdam, Amsterdam 1081 HV, The Netherlands; ^f^Leverhulme Centre for Nature Recovery, University of Oxford, Oxford OX1 3QY, United Kingdom; ^g^Distinguished Fellow, Food and Land Use Alliance- India, New Delhi 110016, India; ^h^School of Environmental Sciences, Jawaharlal Nehru University, New Delhi 110067, India; ^i^Special Centre for Disaster Research, Jawaharlal Nehru University, New Delhi 110067, India; ^j^Betty and Gordon Moore Centre for Science, Conservation International, Arlington, VA 22202; ^k^Conservation International Europe, Brussels 1060, Belgium

**Keywords:** biodiversity, climate change mitigation, spatial prioritization, societal needs, reforestation

## Abstract

Ecosystem restoration is inherently a complex activity with inevitable tradeoffs in environmental and societal outcomes. These tradeoffs can potentially be large when policies and practices are focused on single outcomes versus joint achievement of multiple outcomes. Few studies have assessed the tradeoffs in Nature’s Contributions to People (NCP) and the distributional equity of NCP from forest restoration strategies. Here, we optimized a defined forest restoration area across India with systematic conservation planning to assess the tradeoffs between three NCP: i) climate change mitigation NCP, ii) biodiversity value NCP (habitat created for forest-dependent mammals), and iii) societal NCP (human direct use of restored forests for livelihoods, housing construction material, and energy). We show that restoration plans aimed at a single-NCP tend not to deliver other NCP outcomes efficiently. In contrast, integrated spatial forest restoration plans aimed at achievement of multiple outcomes deliver on average 83.3% (43.2 to 100%) of climate change mitigation NCP, 89.9% (63.8 to 100%) of biodiversity value NCP, and 93.9% (64.5 to 100%) of societal NCP delivered by single-objective plans. Integrated plans deliver NCP more evenly across the restoration area when compared to other plans that identify certain regions such as the Western Ghats and north-eastern India. Last, 38 to 41% of the people impacted by integrated spatial plans belong to socioeconomically disadvantaged groups, greater than their overall representation in India’s population. Moving ahead, effective policy design and evaluation integrating ecosystem protection and restoration strategies can benefit from the blueprint we provide in this study for India.

## Significance

Delivery of ecosystem restoration plans can lead to gains and losses of environmental and societal benefits, disproportionately impacting different groups of society. The tradeoffs and inequity can potentially be large when considering plans focused on a single benefit. Such information is especially lacking in tropical countries, such as India, that must balance local societal needs while delivering actions for ambitious global climate change and biodiversity goals. Here, we show that forest restoration schemes aimed at multiple objectives deliver most of the available benefits, implying minimal tradeoffs. Such schemes deliver benefits evenly across potential restoration areas, implying multiple land options for implementation. Lastly, these schemes are equitable as they deliver benefits to a large proportion of Indians who are socioeconomically disadvantaged.

While forest restoration is sometimes viewed as a simple solution to climate change, it is in practice a complex endeavor (1). There are inherent tradeoffs and inequity in the benefits accrued from restoring forests (2, 3). Global forest restoration action and financial mechanisms aimed at increasing the area of restored forests for climate change mitigation (4) can lead to a carbon-centric approach to decision-making. Such strategies can potentially increase tradeoffs with other desired societal and environmental outcomes (5, 6). Hence, policy makers need to consider how forest restoration strategies can achieve multiple objectives, accounting for potential tradeoffs and inequity issues (7, 8).

Approximately one-third of the global human population live in potential forest restoration areas in the Global South (9). Successful delivery of forest restoration in the Global South requires prioritizing the needs of local communities, and can be enhanced by affording of rights and ensuring local participation in rule-making and community management (9, 10). Indeed, equitable access to resources and benefits helps to alleviate the risks of elite capture, overharvest, and exclusion (11). Nature’s Contributions to People (NCP) has emerged as a useful conceptual framework that allows weaving of social equity and fairness into the design of conservation and restoration actions (12, 13). NCP goes beyond economic valuation of ecosystem conservation and restoration strategies, with emphasis on tangible outcomes to humans (14). Distributional equity, which is the equitable sharing of costs, benefits, rights, and responsibilities is one of four equity aspects that is widely studied in conservation (15). Further, there is a direct link between distributional equity and the other three equity aspects (procedural, recognitional, and contextual) when implementing ecosystem restoration schemes (16), which corroborates the importance of distributional equity. Yet, despite the large potential opportunity for forest restoration in the Global South and the substantial potential impacts on many people, especially those marginalized, equity issues are rarely considered in the design of landscape-scale forest restoration plans (17).

Systematic conservation planning provides a structured multiobjective decision-support framework to identify spatial priorities for conservation and restoration activities in addition to other modeling techniques including partial equilibrium and integrated assessment models. Areas can be identified that deliver multiple objectives in a cost-effective manner while balancing tradeoffs between the objectives (18). Systematic conservation planning has been used to delineate potential priority areas for protection of biodiversity (19), for biodiversity, climate change mitigation, and water resource benefits from conservation (20), and for food production and biodiversity from conservation and restoration (21). It has also been used to quantify spatial tradeoffs between climate change and biodiversity extinction mitigation accounting for costs from forest restoration activities globally (22). However, very few studies include people-centric benefits (such as heritage, nature tourism) with preliminary findings in Europe suggesting that priority areas for conservation when considering people’s values and needs rarely coincide with optimal areas for regulating and material NCP such as climate change mitigation, air quality regulation, flood control, and pollination services (23). Also, though evidence from such studies advance environmental decision-making, these studies tend to overstate one ecosystem service, such as climate change mitigation (24). Last, to the best of our knowledge, there are no studies that use systematic conservation planning to delineate areas for delivery of NCP, that also address distributional equity from forest restoration activities in the Global South.

India is an exemplary case to analyze the above-mentioned lacunae for four important and juxtaposing reasons. First, the country has ambitious targets for forest restoration and climate change mitigation (25), while having limited suitable land area remaining after accounting for existing land uses and covers (26). Second, it has equally ambitious pledges to align environmental and socioeconomic development through programs such as the Aspirational Districts Programme (27) and the National Mission on Biodiversity and Human Well-Being (28). Third, there are diverse ecologically and evolutionarily important and distinct species assemblages, ecosystems, biogeographic zones, and biodiversity hotspots across India. Fourth, though the extent of poverty has declined in India, regional wealth inequality continues (29). In this context, 85% of priority areas delivering multiple ecosystem services (water- and climate change mitigation-related), habitats, and biodiversity are outside of India’s protected area networks (30). Also, many of India’s poorest subjurisdictions have high potential for climate change mitigation through restoration, presenting opportunities to address poverty while delivering on climate change mitigation goals (31). Hence, complementary information incorporating tradeoffs in multiple objectives and equity issues can support sustainable and equitable policy and decision-making related to forest restoration for multiple societal and environmental objectives.

Here, we combine the frameworks of NCP and systematic conservation planning to evaluate the tradeoffs in NCP from forest restoration that focus on a single-objective/NCP versus integrated forest restoration plans that are aimed at joint achievement of multiple objectives/NCP. The three NCP of focus are 1) regulating NCP of climate change mitigation potential, henceforth climate NCP, 2) regulating NCP of forest habitat created for forest-dependent mammals, henceforth biodiversity value NCP, and 3) human direct use of nature for livelihoods, housing construction material, and energy, henceforth societal NCP. Climate NCP is quantified as the carbon sequestered in the first 30 y of natural forest regrowth. Biodiversity NCP is quantified as the proportion of mammal species for which the habitat target can be met by restoration, where the target is the minimal additional area of habitat (AOH) required by the mammal to be listed as “Least Concern” in 10 y or three generations, whichever is longer. Societal NCP is the average number of people in restoration areas reliant on forests across the three needs (see *Methods* and *SI Appendix* for further details).

**Fig. 1.**
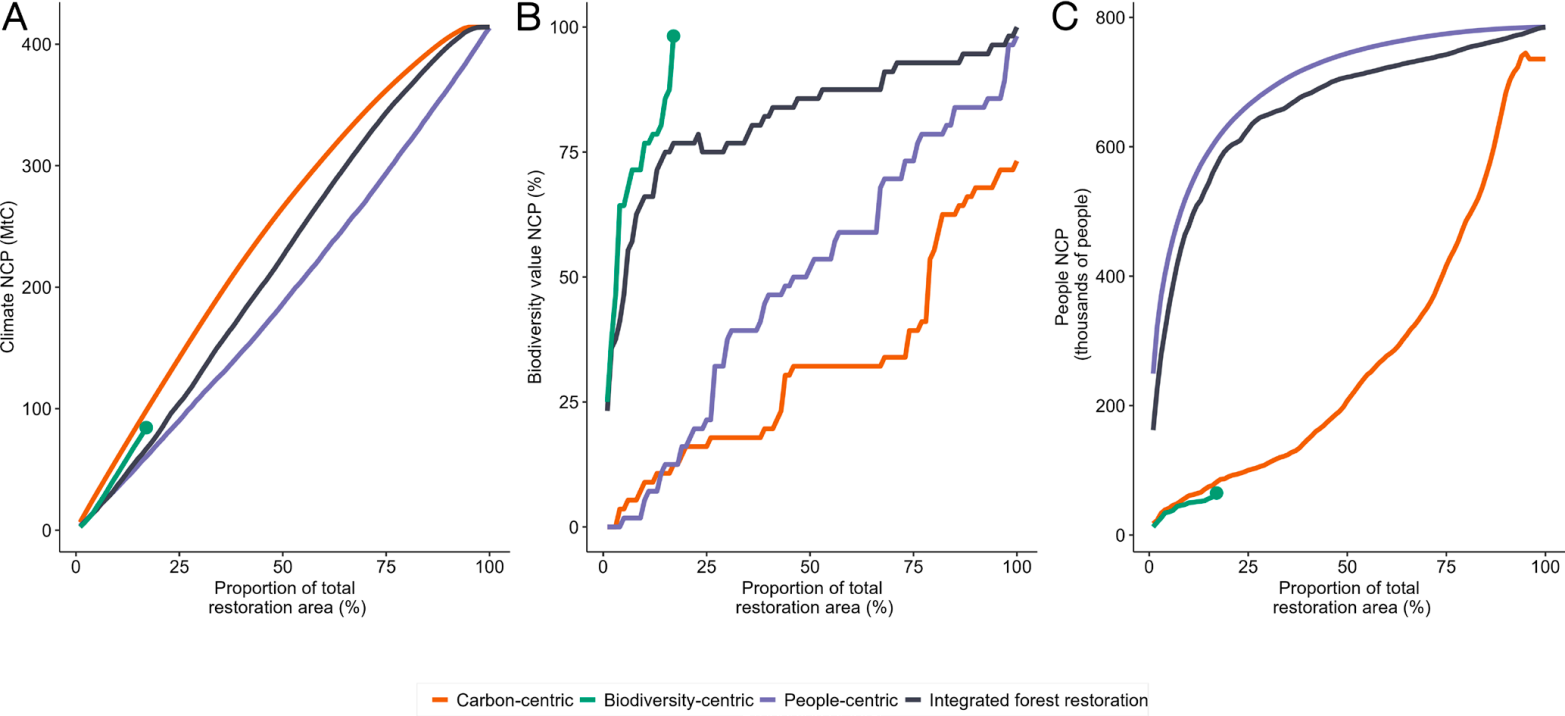
NCP accumulation curves across restoration area (3.88 Mha; *SI Appendix*, Fig. S1) for (*A*) climate NCP, (*B*) biodiversity value NCP and (*C*) societal NCP. Single-objective plans such as the carbon-centric plan provide the highest amount of their target NCP for a given budget across all plans. Integrated forest restoration plans are the next best solution for biodiversity and societal NCP, and for climate NCP above 21% of the restoration area. Integrated plans provide most of the respective NCP in all cases, implying the least tradeoffs [on average 83.3% (43.2 to 100%) of maximum climate NCP (*A*), 89.9% (63.8 to 100%) biodiversity value NCP (*B*), and 93.9% (64.5 to 100%) societal NCP (*C*)]. Irrespective of plan type, there is a near-linear increase in accumulation of climate NCP with a linear increase in restoration area in which a plan is implemented (*A*). Accumulation of societal NCP from an integrated plan follows a concave down increasing trend (*C*). Note that for the biodiversity-centric plan, all biodiversity value NCP is achieved within 17% of the restoration area if the plan were to be implemented and hence the spatial optimization automatically ceases [indicated by the green dot in the biodiversity-centric plan trends (*A*–*C*)]. This would imply after restoring 17% of the identified restoration area as per the biodiversity-centric plan, areas prioritized by other plans can be implemented to deliver remaining NCP.

**Fig. 2.**
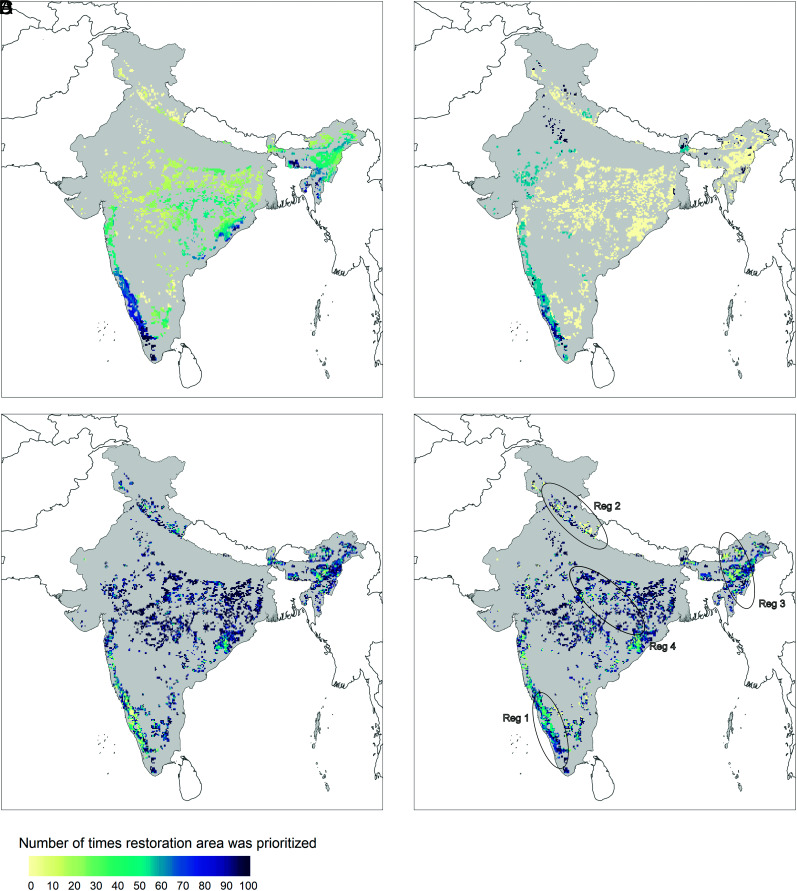
Spatial patterns in optimal restoration area across 3.88 Mha (colored area) when implementing (*A*) climate-centric, (*B*) biodiversity-centric, (*C*) people-centric, and (*D*) integrated plans. There is high concentration of focal restoration areas for carbon-centric plans in the Western Ghats along the western coast, north-eastern Indian, and northern sections of the Eastern Ghats on the eastern coast (shades of blue in *A*). The biodiversity-centric plan delineated the Western Ghats and the restoration opportunity toward the northwest as optimal (shades of blue in *B*). There is a more evenly distributed pattern of focal areas for societal NCP when implementing a people-centric plan (*C*) and for integrated plans (*D*), implying multiple options for site selection. Certain regions were identified to be optimal only in the integrated plan and not the people-centric plan (Reg 1) and vice versa (Reg 2 and Reg 3 in *D*). All areas in gray were the initial study area for the analyses and areas in white were not included in the analyses.

**Fig. 3.**
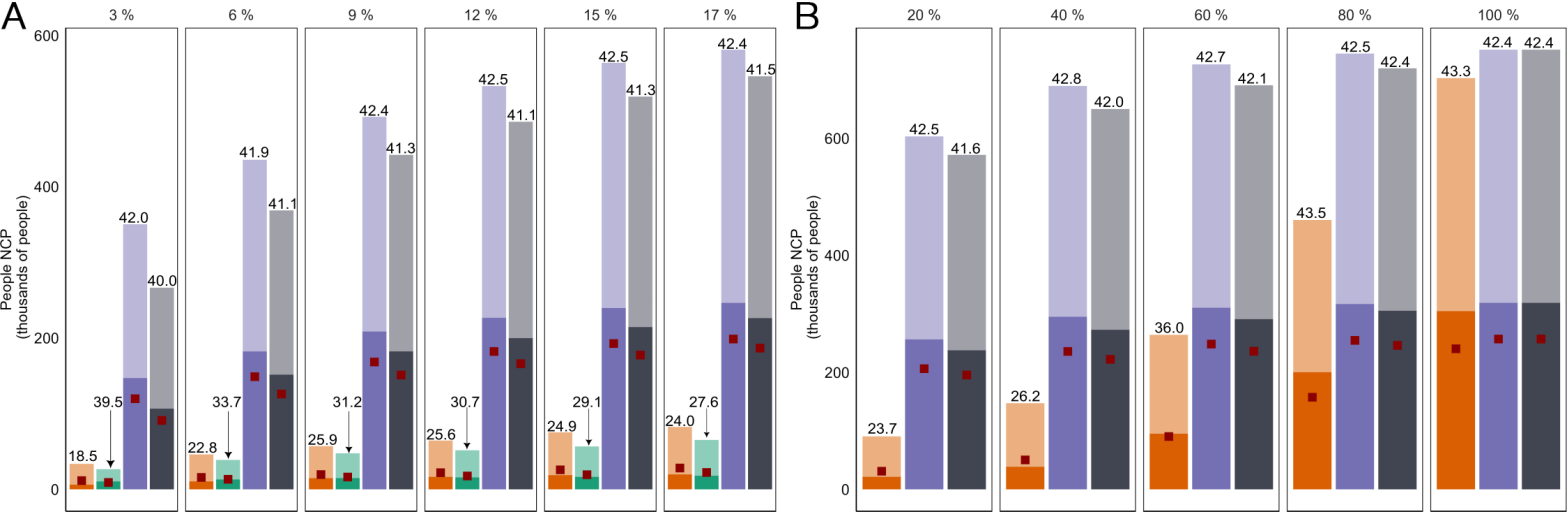
Distributional equity in the NCP delivered between plans. Panel (*A*) compares plans within 17% of the restoration area in which biodiversity-centric plans are optimized. Panel (*B*) compares remaining plans beyond 17% of the restoration area in which the carbon-centric, people-centric, and integrated forest restoration plans continue to deliver remaining NCP. The proportions of restoration area at which distributional equity is calculated are mentioned at the *Top* of each subpanel. The height of each bar represents the average total societal NCP delivered as shown in Fig. 1*C*. The shaded portion of each bar represents the societal NCP delivered to those who are socioeconomically disadvantaged. The dark red colored square in each bar represents the national average statistic of proportion of socioeconomically disadvantaged people in India (34.2% of each bar). The number above each bar represents the proportion of the total people to whom societal NCP is delivered by each forest restoration plan who are socioeconomically disadvantaged. Across any increment of restoration area in which a people-centric plan is implemented, the highest number of people who are socioeconomically disadvantaged are benefited (purple). A similar pattern is found when implementing an integrated plan (gray). However, the carbon-centric plan has a below-average benefit to socioeconomically disadvantaged people up to nearly 56% of the restoration area (orange). The biodiversity-centric plan (green) benefits a consistently below-average proportion of socioeconomically disadvantaged people.

In this study, we use mixed integer linear programming optimization (MILP) technique to apply the systematic conservation planning framework (20–22), using spatial information about climate change mitigation potential, mammal species’ habitat, and elevational preferences and people’s reliance on forests for basic needs in conjunction with socioeconomic data of the Indian population. We analyze four types of plans: carbon-centric, biodiversity-centric, and people-centric plans (i.e., single-NCP plans), and integrated forest restoration plans aimed at joint achievement of multiple NCP, henceforth integrated plans. We answer three questions:

(1) What are the trends in NCP from forest restoration plans aimed at a single-NCP versus integrated plans? We present these results as NCP accumulation curves for incremental increase in restoration area over which plans are implemented (*Results* and Fig. 1).
(2) What are the spatial patterns in the selected restoration areas from single-NCP versus integrated plans? We present these results as the frequency with which an area is included in the solution, as the area restored is increased from 1 to 100% (in increments of 1%) of the maximum potential restoration area (*Results* and Fig. 2).
(3) What is the distributional equity of societal NCP from single-NCP versus integrated plans? We present and discuss these results as the fraction of socioeconomically disadvantaged people and women to whom societal NCP will be delivered (*Results* and Fig. 3).

We answer these research questions for a potential restoration area of 3.88 Mha across India, henceforth the restoration area. Spatially explicit MILP optimization is a mathematical formulation describing the problem of selecting the best area configuration that generates maximum NCP, while meeting constraints of the total restoration area available and the minimum value of the targeted NCP (18). Hence, the results consist of the optimal restoration area in which multiple NCP (in case of the integrated forest restoration plans) or a single-NCP (in case of the carbon, biodiversity, and people-centric plans) delivered is the maximum possible within the formulated constraints (*SI Appendix*, Fig. S1; see Workflow in *SI Appendix*, Fig. S2, *Methods* and *SI Appendix* for additional details).

## Results

### Integrated Forest Restoration Plan Delivers on All Three NCP without Major Individual Compromises.

The carbon-centric plan delivered the highest climate NCP (6.4 to 414.1 MtC across 1 to 100% of restoration area). Up to 5% of the total restoration area, the next highest amount of climate NCP delivered fluctuated between the people-centric and integrated plans. The biodiversity-centric plan offered the next best delivery of climate NCP, on average 77.6% (62.7 to 85.8%) of the carbon-centric strategy, in the 5 to 21% range of restoration area. However, if more than 21% of the restoration area was restored, the integrated plan delivered the next highest climate NCP, on average 88.8% (73.4 to 100%) of the carbon-centric plan. The people-centric plan delivered the least climate NCP across the restoration area, on average delivering 72.8% (55.4 to 99.8%) of the carbon-centric plan (Fig. 1*A*). Note that the carbon-centric plan does not deliver 100% of any of three NCP because 4.6% of the restoration area does not have information about carbon sequestration yet were included assuming that these areas deliver biodiversity and societal NCP (*SI Appendix*, Fig. S4; see *Methods* and *SI Appendix*).

The biodiversity-centric plan delivered 100% of the attainable biodiversity value NCP within 17% of the restoration area, i.e., no further accumulation of biodiversity NCP was possible, such that the spatial optimization algorithm automatically ceased. This is because of how biodiversity value NCP was calculated: Of all forest-dependent mammals in India, we considered only the 56 for which there would be a significant additional amount of target habitat created by forest restoration (*Methods* and *SI Appendix*). The remaining plans showed a steady increase in the proportion of the mammals for which the biodiversity value NCP is met. At 17% of the restoration area, integrated plan delivered biodiversity value NCP to 76.8% of the mammals, while carbon-centric and people-centric plans delivered biodiversity value NCP to 12.5% of the mammals (Fig. 1*B*). Overall, it takes 5.8 times more area for the integrated and carbon-centric plans to achieve the same biodiversity outcomes as the biodiversity-centric plan. The high efficiency of the biodiversity-centric plan suggests that another plan could be implemented to accumulate the remaining NCP beyond 17% of the restoration area. People-centric plans do not achieve 100% of any of the three NCP because 0.15% of the restoration area does not have information about the societal NCP. However, like the carbon-centric plan, we assume these areas will deliver carbon and biodiversity value NCP.

For societal NCP, the people-centric plan delivered societal NCP to many people over relatively small amounts of restoration area (a downward concave increasing trend delivering societal NCP to 0.3 to 0.8 million people across 1 to 100% of restoration area). The integrated plan followed a similar trend delivering societal NCP to on average of 93.9% (64.9 to 100%) of the people-centric plan. In contrast, the carbon-centric plan delivered societal NCP to few people over relatively large amounts of restoration area (concave upward increasing trend delivering societal NCP to an average of 37.5% (6.9 to 94.9%) of the people-centric plan), highlighting a clear tradeoff between plans focused on climate NCP and societal NCP. Last, the biodiversity-centric plan delivered societal NCP to the least number of people, on average 8.6% (5 to 10.6%) of the people-centric plan within 17% of the restoration area (Fig. 1*C*).

Overall, the integrated forest restoration plan delivered on average 83.3% (43.2 to 100%) of maximum climate NCP, 89.9% (63.8 to 100%) biodiversity value NCP, and 93.9% (64.5 to 100%) societal NCP. The majority of climate NCP was delivered irrespective of the type of plan, whereas only a minority of biodiversity and societal NCP were delivered by single-objective plans not focused on that NCP.

### Integrated Restoration Plan Results in More Even Restoration Area Selection as Opposed to a Clustered Pattern from Single-NCP Plans.

We found clear differences in the spatial patterns of NCP delivery from the four plans. Optimal restoration areas clustered in two Indian biodiversity hotspots (32) for delivery of climate and biodiversity value NCP: The Western Ghats along the western coast of the peninsula was selected for both biodiversity-centric and carbon-centric plans, while North-eastern India, another biodiversity hotspot, along with the northern section of the Eastern Ghats on the eastern coast were also foci for implementation of the carbon-centric plan. The carbon- and biodiversity-centric plans showed contrasting east-west spatial trends, with the carbon-centric plan being pivotal in the Central Highlands and Eastern Ghats of central India and the biodiversity-centric plan selecting sparse restoration area in northwest India (Fig. 2 *A* and *B*). The people-centric and integrated plans resulted in a relatively more even pattern of NCP delivery across the restoration area (Fig. 2 *C* and *D*). For the integrated plan, the restoration area in the southern section of the Western Ghats (Reg 1 in Fig. 2*D*) and parts of north-eastern India (Reg 3 in Fig. 2*D*) were more frequently selected compared to the restoration area in the north (Reg 2 in Fig. 2*D*). Furthermore, the restoration areas identified by the people-centric and integrated plans differed in parts of central India (Reg 4 in Fig. 2*D*).

### Integrated Forest Restoration Plans Deliver Maximal Equity of Societal NCP to Socioeconomically Disadvantaged People and Women When Compared to the National Average.

People-centric plans consistently delivered societal NCP to the greatest number of people that are socioeconomically disadvantaged [average 42.5% (40.5 to 42.8%) of the total number of people to whom societal NCP is delivered across 1 to 100% of the restoration area]. The integrated plan also delivered societal NCP consistently to an average of 41.9% (39.8 to 42.6%) of the total number of people who are socioeconomically disadvantaged. Contrastingly, the percentage of socioeconomically disadvantaged people benefited by the carbon-centric plan averaged 32.5% (15.1 to 43.9%) (Fig. 3*B*). Within 17% of the restoration area, 33.3% (27.6 to 44.6%) of the total people to whom societal NCP is delivered by the biodiversity-centric plan are socioeconomically disadvantaged (Fig. 3*A*). Overall, people-centric and integrated forest restoration plans had above-average equity across the restoration area when compared to the national average proportion of socioeconomically disadvantaged people at 34.2%. The carbon-centric plan initially benefited a small fraction of socioeconomically disadvantaged people but became more equitable as the restored area increased. The biodiversity-centric plan delivered societal NCP to a below-average proportion of socioeconomically disadvantaged people. The average proportion of total people to whom societal NCP are delivered that are women was consistent across all four plans (49.1% for the integrated plan, 50.2% for the carbon-centric plan, 49.9% for the biodiversity-centric plan, and 49.1% for the people-centric plan), with all plans delivering above-average societal NCP to women when compared to the national average of 48.9% (*SI Appendix*, Fig. S3).

## Discussion

Sustainable and equitable policy design and decision-making for forest restoration requires evidence of the tradeoffs between multiple societal and environmental benefits and the equity of such benefits across society. Here, considering the case of India, we determine the utility of forest restoration spatial plans aimed at delivery of multiple NCP across a defined area of 3.88 Mha, potentially suitable for forest restoration (restoration area; *SI Appendix*, Fig. S1). Overall, we find that forest restoration plans focused on a single-NCP can result in tradeoffs with other NCP. For example, a carbon-centric plan does not necessarily deliver benefits to biodiversity or people. In contrast, an integrated forest restoration plan aimed at joint achievement of multiple outcomes, on average, can deliver a majority of the three NCP considered in this study, implying the least tradeoffs between NCP. Such a plan prioritizes multiple restoration areas that can deliver similar NCP, thereby providing multiple options of sites for implementation of the plan. Lastly, an integrated forest restoration plan and a people-centric plan deliver livelihoods, energy, and housing construction material (collectively societal NCP) to a disproportionately high fraction of people belonging to socioeconomically disadvantaged groups.

For a given area of restoration, integrated plans deliver a majority of available NCP across the restoration area in India (Figs. 1 and 2*D*). This is because integrated plans identify wide and evenly distributed restoration area by accounting for widely distributed forest resources such as fuel wood and timber for housing construction. Indeed, these key results corroborate global analyses showing that integrated conservation and restoration strategies can simultaneously provide human needs (food) while continuing to provide biodiversity benefits (21). Thus, the overall evenly distributed pattern of delivery of NCP by integrated plans (Fig. 2*D*) provide flexibility in site selection for implementation of forest restoration action. We argue that this flexible characteristic of integrated forest restoration plans is especially useful in policy design. More site options in which NCP is delivered could potentially ensure that benefits and costs of restoration are more fairly distributed, and lead to wider engagement of stakeholders and policy actors in implementation (33). However, in specific regions in central India, integrated forest restoration plans do not deliver the maximum possible NCP considering the constraints of total restoration area and targets (shades of green and yellow in Reg 4 in Fig. 2*D*). These areas contrast with the findings of Srivathsa et al. (30) due to the differing criteria of biodiversity and water benefits considered and the differing goal of finding priority areas for ecosystem protection rather than restoration. Since the objective of our study was comparison of forest restoration plans focused on a single-NCP and multiple NCP, our biodiversity criteria focused on a subset of forest-dependent mammals that would benefit from forest habitat area restoration. Nevertheless, the same parts of central India are identified in people-centric forest restoration plans in our study, and align with evidence that subjurisdictions in central India can be targeted for poverty alleviation while continuing to mitigate climate change from forest restoration activities (31). Hence, we recommend that further contextualization of the restoration area identified/not identified for implementation of integrated forest restoration plans is needed. Such contextualization can be done by including information about the potential participation of local and indigenous communities in restoration programs (9) and existing governance and land tenure regimes (10, 31, 34).

Our study provides vital information about the distributional equity of NCP from forest restoration plans. Particularly, we show that integrated plans consistently distribute NCP of livelihoods, energy, and housing construction material (societal NCP) above the national average proportion of people belonging to socioeconomically disadvantaged sections of Indian society (Fig. 3). This finding is important because it supports the implementation of one of the core visions of the United Nations Decade on Ecosystem Restoration (16, 35). Indeed, our results of distributional equity of different restoration programs across the restoration area can be used to inform strategic planning of capacity-building programs to support marginalized groups (36). Caste, one important attribute denoting India’s socioeconomic societal groups, has been shown to be an influential factor when mobilizing savings (37), farm landowner’s choices for environmental management action (38) and implementation of community-based climate change adaptation strategies in Indian villages (39). Hence, findings from this study that integrated plans deliver maximum NCP to the highest number of Indians belonging to socioeconomically disadvantaged communities relative to other types of forest restoration plans can support evidence-based equitable policy formulation.

There is a gendered perspective to distributional equity. Our results suggest that all types of forest restoration plans deliver societal NCP to women and men equally in India (*SI Appendix*, Fig. S3). However, region-specific evidence shows that the primary responsibility of fuelwood collection for energy (cooking and heating) lies on women (40–42). Similarly, women’s role in collection and sale of nontimber forest products has been considered a potential avenue of income and empowerment of women (43). Hence, incorporating information about the extent to which the burden and benefits of resource access from forests falls on women would help to refine our estimates. Furthermore, we recommend an intersectional lens considering gender, age, race, class, and power dynamics (13, 16) to better understand the distributional and other equity aspects from forest restoration plans.

We found an overall tradeoff between biodiversity and climate NCP from carbon-centric forest restoration plans (Fig. 1 *A* and *B*), while biodiversity-centric plans tend to deliver climate NCP. We posit that this is because our biodiversity metric included only mammals for which there would be significant additional forest habitat created from forest restoration in the restoration area. Hence, while most restoration areas with high biodiversity value NCP also contained high carbon value (Fig. 2 *A* and *B*), many areas with high carbon value did not overlap with our target species ranges. However, biodiversity-centric plans also prioritized some semiarid regions of north-western India, which do not have high carbon potential. Indeed, inclusion of a single biodiversity metric, in our case restoration area targets, results in a more limited restoration area being selected than if multiple biodiversity criteria were used such as extinction risk (22), functional and phylogenetic diversity (44). Nevertheless, there is a synergy in climate and biodiversity value NCP from biodiversity-centric and carbon-centric plans in the 5 to 21% range of restoration area (Fig. 1 *A* and *B*). This reflects the selection of certain biodiversity strongholds, such as the Western Ghats, restoration of which can also provide high carbon sequestration rates. Such irreplaceable forest ecosystems should be continued to be protected, while engaging in targeted forest restoration, especially along species movement corridors (45).

People-centric plans lead to tradeoffs with both climate and biodiversity NCP (Figs. 1*C* and 2*C*). Similar to findings about cultural NCP in Europe (23), societal NCP is delivered across wide and disaggregated spans of restoration area. We hypothesize that the widely distributed societal NCP reflects the high dependence on forests and other natural resources for basic needs across many parts of rural India. 65% of India’s total population still live in rural areas (46) with potentially high reliance on nature for key needs. For example, India accounts for 35% of the world’s population with no continuous access to electricity (47), implying reliance on alternative sources such as fuelwood. Hence, it would be worth validating our findings by considering the spatial distribution of the supply and demand of key basic needs (48). Additional high-resolution and disaggregated information about the employment and income generation from natural forests (49) could further refine our results about societal NCP delivered from different forest restoration plans.

Overall, in the UN Decade on Ecosystem Restoration there is an urgent need to develop policy and practice toward a shared future for nature and people. Considering the case study of India, here we provide a blueprint to understand the implications of different policy types when planning for forest restoration, an important strategy for tackling environmental and societal challenges (7). We show that forest restoration policies that integrate multiple environmental and societal outcomes can potentially deliver most of the possible benefits for a given area restored, resulting in the least tradeoffs. Since multiple optimal locations of restoration areas deliver the same NCP from integrated forest restoration plans, context-specific information can be included for on-the-ground implementation activities, thereby balancing local needs and global benefits. Our prototype methodology of combining systematic conservation planning, specifically spatial optimization, and equity analyses provides an approach that is more transparent, finer resolution, and more readily adapted to local context and policy goals than coarse scale economic modeling such as partial equilibrium and integrated assessment models (50). Indeed, land use policy and planning in India needs to focus on the delivery of multiple ecosystem benefits encompassing environmental and societal goals when assessing available land options for implementation of forest restoration programs. To restore multifunctional landscapes while meeting different societal needs across socioeconomic groups in India, we recommend formulation of a dedicated multiobjective ecosystem restoration policy within the existing framework of national policies that include the National Afforestation Programme 2002, Urban Forest Scheme 2020, and the Agroforestry Policy 2014 (51). With the increasing need for land use strategies that tackle multiple challenges, especially that of climate change (52, 53), effective policy design and evaluation integrating protection and restoration of natural ecosystems will benefit from the blueprint we provide in this study.

## Methods

We mapped the potential forest restoration area, henceforth restoration area, as the area suitable for naturally regenerating native forests at 10 × 10 km spatial resolution. We followed ref. 26 to account for 1) biophysical conditions suitable for a forest biome and 2) current land uses and covers that are unsuitable for natural forest regeneration, while protecting food supply and meeting additionality requirements for emissions reductions (see *SI Appendix*, Table S2 for information about data used). Consequently, the area remaining after exclusion of above-mentioned land uses and covers was 3.88 Mha across India (*SI Appendix*, Fig. S1).

We determined the climate, biodiversity value, and societal NCP that will be generated if the restoration area of 3.88 Mha were to naturally regenerate into native forests. First, using information about carbon sequestration rates of native forests from ref. 54, we estimated the above and belowground carbon stock accumulation for the first 30 y of natural forest regrowth in the restoration area. There is no information of above and/or belowground carbon sequestration rates from ref. 54 in the Himalayan subtropical pine forests, Western Himalayan broadleaf forests, xeric areas of western India, and the Deccan plateau, approximating to 4.6% of the restoration area of 3.88 Mha. These restoration areas were included assuming delivery of biodiversity value and societal NCP. The climate change mitigation potential of restoring natural forest cover in the whole area i.e. climate NCP was 414.1 MtC (28.7 tC to 1.3 MtC in a 10 km × 10 km restoration area) (*SI Appendix*, Fig. S4).

Second, we calculated biodiversity value NCP as the number of forest-dependent mammal species for which restoration area targets could be met. We focused on the subset of 56 species for which restoration would result in significant additional habitat. First, we calculated the potential AOH for each species based on the preferences of forest habitat type and elevational limits (55) that fall on and outside the restoration area within each of the mammals’ ranges. Second, we followed a twofold process to calculate the target restoration area to be restored for each species. This protocol of setting target areas was inspired by the International Union for Conservation of Nature (IUCN) Red List assessment protocol, which identifies the minimum AOH needed to be listed as Least Concern in 10 y or three generations (whichever is longer). We calculated a preliminary AOH restoration target for each species by adapting the area-based target protocol from refs. 20 and 21 using the potential and current AOH (Table 1). Current AOH is assumed to be the potential AOH not identified as a restoration area. From the preliminary target, we then deducted the current AOH to arrive at a restoration target. The total area delivering biodiversity value NCP was 1.46 Mha [0.79 ha for Mandelli's mouse-eared bat (*Myotis sicarius*)—0.42 Mha for Peyton’s whiskered bat (*Myotis peytoni*)] (*SI Appendix*, Table S1). We quantify biodiversity value NCP as the proportion of the 56 mammals for which the AOH target is met.

**Table 1.** Target setting protocol used to calculate preliminary target restoration area required by each species

Potential AOH	Current AOH	Preliminary target
<2,200	<2,200	Potential AOH
<2,200	>2,200	min [max (2,200, 0.8*potential AOH), 10^6^]
>2,200	>2,200	min [max (2,200, 0.8*potential AOH), 10^6^]

Potential AOH and current AOH (defined as potential AOH outside the restoration area) were calculated within the species ranges of each mammal. The unit of all variables in the table is km^2^.

Third, we calculated the societal NCP as the number of people who directly rely on forests for their livelihoods, energy, and housing construction material across the restoration area from ref. 56. We used a simple weighting based on ref. 57 to account for rural-urban population reliance and for livelihoods we considered only those whose primary occupation is not agriculture and industry sectors using information from ref. 58. The number of people reliant on forests for livelihoods, energy, and housing construction material are 0.28 million, 1.58 million, and 0.48 million, respectively. 0.15% of the restoration area did not have societal NCP due to lack of data about the rural-urban reliance (*SI Appendix*, Fig. S5). We further discuss societal NCP as the average number of people reliant on forests for the three needs.

To estimate distributional equity in societal NCP, we extracted the percentage of the total population that identify as women and the percentage of the total population that belong to Scheduled Castes and Tribes, the official designation of Indians belonging to socioeconomically disadvantaged communities in India (59), in 5,881 Indian subjurisdictions for which socioeconomic information is available from ref. 58.

We explore tradeoffs in NCP from plans aimed at single-NCP and integrated forest restoration plans aimed at multiple NCP by assessing optimal restoration area selections for each plan. Such an optimal area selection is done through MILP optimization for each 1% increment until the entire restoration area of 3.88 Mha is attained. We used the *prioritizeR* R package (60) to implement the MILP optimization. We formulated the problem as the minimization of the weighted proportional summed shortfall of NCP delivery *y_j_* [difference between the NCP target (*t_j_*) and the NCP delivered when a particular amount of restoration area is “grown”, where the NCP target is the biodiversity value NCP and the maximum possible NCP for the climate and societal NCP *J_j_*where *J* is an NCP and *j* is the amount of the NCP *J* in the restoration area] for a given budget of restoration area *I_j_* (1 to 100% of the 3.88 Mha) (Eqs. **1** and **2**).

[1] $$\mathrm{Minimize}\sum_{j\;=\;1}^{J}w_{j}\frac{y_{j}}{t_{j}},$$

subject to

[2] $$\sum_{i\;=\;1}^{I}x_{i}r_{\mathrm{ij}}+y_{j}\geq t_{j}\forall j\in J,$$

where we implemented a proportional decision variable *x_i_* which is the proportion of the restoration area *i* to be identified (proportion between 0 and 1, where 0 means the restoration area is not identified for implementation of plan and 1 means vice versa), such that all the proportional areas (*c_i_* 1% increments) do not exceed the total restoration area of 3.88 Mha (B) (Eq. **3**)

[3] $$\sum_{i\;=\;1}^{I}x_{i}c_{i}\leq B.$$

We considered four types of forest restoration plans: i) carbon-centric plans, ii) biodiversity-centric plans, iii) people-centric plans, and iv) integrated forest restoration plans. For the first three types, we excluded all other NCP by weighting (*w_j_* in Eq. **1**) only the NCP targeted by the plan; for the integrated forest restoration plans, we weighted all NCP equally. In total, we solved 400 optimization problems (four plans* 100 runs for each of the 1% increments of restoration area). This resulted in four nested sets of priorities such that trends in NCP delivery was assessed using NCP accumulation curves with increasing restoration area.

## Supplementary Material

Appendix 01 (PDF)

## Data Availability

Geo spatial files of optimized locations for delivery of NCP by different types of forest restoration plans analyzed data have been deposited in Zenodo (10.5281/zenodo.10676360). Information about the ground control points of natural forests across India used to map the potential restoration area has been provided under a Memorandum of Understanding from the publication cited as ref. 61. Previously published data were used for this work (54, 56–58).
