# Supplementary material for: Optimizing restoration: A holistic spatial approach to deliver Nature’s Contributions to People with minimal tradeoffs and maximal equity
Source: Proc Natl Acad Sci U S A. 2024 Aug 12;121(34):e2402970121. doi: 10.1073/pnas.2402970121 (PMC11348303; doi:10.1073/pnas.2402970121)
Supplement: Supplementary file 1 — Appendix 01 (PDF) [file pnas.2402970121.sapp.pdf]

Optimizing Restoration: a holistic spatial approach to deliver Nature's Contributions to People with minimal tradeoffs and maximal equity

Trisha Gopalakrishna[1,2,3*], Piero Visconti[2], Guy Lomax[1,4], Esther Boere[5,2], Yadvinder Malhi[3,6], Parth Sarathi Roy[7], Pawan K. Joshi[8,9], Giacomo Fedele[10,11], Ping Yowargana[2]

[1] Department of Geography, University of Exeter, Exeter EX4 4QE, United Kingdom
[2] International Institute of Applied Systems Analyses (IIASA), Schlossplatz 1 | A-2361 Laxenburg, Austria
[3] Environmental Change Institute, School of Geography and the Environment, University of Oxford, Oxford OX1 3QY, United Kingdom
[4] Global Systems Institute, University of Exeter, Exeter EX4 4QE, United Kingdom
[5] Department of Environmental Geography, Instituut voor Milieuvraagstukken, Vrije Universiteit Amsterdam, 1081 HV Amsterdam, The Netherlands
[6] Leverhulme Centre for Nature Recovery, University of Oxford, Oxford OX1 3QY, United Kingdom
[7] Distinguished Fellow, Food and Land Use Alliance- India, Hyderabad, India
[8] School of Environmental Sciences, Jawaharlal Nehru University, New Mehrauli Road, New Delhi 110067, India
[9] Special Centre for Disaster Research, Jawaharlal Nehru University, New Mehrauli Road, New Delhi 110067, India
[10] Betty and Gordon Moore Centre for Science, Conservation International, Arlington VA, USA
[11] Conservation International Europe, Brussels, Belgium

Email Addresses

Trisha Gopalakrishna - t.gopalakrishna@exeter.ac.uk
Piero Visconti - visconti@iiasa.ac.at
Guy Lomax - g.lomax@exeter.ac.uk
Esther Boere - e.j.m.boere@vu.nl
Yadvinder Malhi - yadvinder.malhi@ouce.ox.ac.uk
Parth Sarathi Roy – psroy13@gmail.com
Pawan K. Joshi - pkjoshi@mail.jnu.ac.in
Giacomo Fedele - gfedele@conservation.org
Ping Yowargana - yowargan@iiasa.ac.at

Supplementary Material

Supplementary Methods

1. Determination of restoration area

(a) Modelling the biophysical envelope of native forests at the pan-India scale

We used a random forest algorithm using information about the location of presences of different forest types and the environmental covariate information at these points as per Gopalakrishna et al., 2022 to determine the biophysical envelope of native forests across India. First, we extracted the information of 11 environmental predictors (Table S2) for 10756 GPS- gathered points of presences of different forest types from Roy et al., 2015. We generated 22453 pseudoabsences or background sites as random points where there is no current forest cover as per Barbet-Massin et al., 2012 and extracted information of the 11 environmental predictors at these background sites (Fig S6). Second, we used 830 trees, 6 nodes at each tree and 3 features to be considered at each node in the random forest algorithm, resulting in the probability of occurrence of native forests. Third, we parameterized the algorithm by assessing the Area under the Curve metric (AUC) by spatial repeated cross validation (0.702) after comparing it to the AUC from repeated cross validation (0.930) (Fig S7). In both repeated cross validation and spatial repeated cross validation analyses, we used 10 repetitions of 5 partitions of the data accounting for the variance introduced due to data partitioning (4). Fourth, to assess the performance of the algorithm, we calculated 10 accuracy metrics- false positive and negative rates, mean misclassification error, Kappa statistic, total positive and negative rates, accuracy, area under curve, balanced accuracy and balanced error rate (Fig S8). Lastly, we used the threshold of 0.494 as the presence threshold corresponding to the least mean misclassification error of 0.069, resulting in 54.58 Mha of the biophysical envelope.

(b) Sequential exclusion of all land uses and covers that cannot be restored to forests

From the biophysical envelop, we sequentially excluded all land uses and covers that cannot be restored to forests. Using Buchhorn et al., 2020, we excluded areas classified as unknown, urban/built-up, bare/sparse vegetation, snow & ice, permanent water bodies, herbaceous wetlands, moss & lichen, oceans & seas and cultivated and managed vegetation/ agriculture. Furthermore, we used an India specific land use-cover dataset from Roy, et al., 2015 (year 2005) to exclude all deciduous & evergreen broadleaf forests, deciduous & evergreen needle leaf forests, mixed forests and grasslands. This resulted in the restoration area of 3.88 Mha (Fig S1).

2. Estimation of Nature's Contribution to People in restoration area

(a) Climate NCP

We calculated the climate NCP as the change mitigation potential resulting from the naturally regenerating total above and belowground carbon stocks in the restoration area (Fig S4; Equation 1). We used aboveground and belowground sequestration rates as per Cook-Patton et al., 2020, which was estimated as a function of 66 environmental covariates and over 13000 georeferenced measurements of carbon accumulation for the first 30 years of natural forests regrowth globally. For India, the aboveground carbon sequestration rate has a range of 0.50 - 4.51 $MgCha^{-1}yr^{-1}$ and the belowground carbon sequestration rate has a range of 0.19 - 2.47 $MgCha^{-1}yr^{-1}$.

$$climate\ NCP\ (MtC) = above + belowground\ carbon\ sequestration\ (MtCha^{-1}yr^{-1}) * restoration\ area\ (ha) * 30\ years$$

(Equation 1)

(b) Biodiversity value NCP

First, we extracted the species ranges of all mammals from the within the Indian jurisdictional boundary whose habitat type is forest as per the IUCN Red List of Threatened Species database (8) (n=350). Second, we considered all mammals for which there is information available about its habitat preferences (forest type) and elevational limits in the IUCN Red List of Threatened Species database (n=291). However, a key challenge lies in estimating potential forest habitat preferences of a mammal using contemporary habitat type spatial information. Hence, for each mammals' species range, we determined the potential forest habitat preference by assigning restoration area in the range to be the nearest forest habitat type as mapped in (9) by assuming maximum probability of natural regeneration from nearby seed sources (10). Third, for each of the 291 mammals we estimated the potential area of habitat (AOH) as per Brooks et al., 2019 by using the suitable potential forest habitat type and elevational limits.

Fourth, we followed a twofold process to calculate the target restoration area required by each mammal. This protocol of setting target areas are inspired by the IUCN Red List assessment protocol, and identify the AOH that have a species must have to be listed as 'Least Concern' in 10 years or 3 generations (whichever is longer): (i) we calculated a preliminary target by adapting the area-based target protocol from (12) and (13) using the potential AOH and the potential AOH outside the restoration area for each mammal (see Table 1) and (ii) from this preliminary target, we deducted the potential AOH outside the restoration area, resulting in the target restoration area required i.e. biodiversity value NCP. Of the 291 mammals, 235 have potential AOH outside the restoration area exceeding the target area needed. Hence, we considered remaining 56 mammals for which forest restoration would result in significant additional forest habitat. Of these species, 16%, 7% and 1.7% are classified as endangered, near threatened and critically endangered, respectively, 10.7% are listed as vulnerable and data deficient and the remainder are classified as least concern by the Red List Assessment (Table S1).

(c) Societal NCP

We calculated societal NCP as the product of the population density from Center for International Earth Science Information Network - CIESIN - Columbia Univeriisty, 2018 and the percentage societal reliance from Fedele et al., 2021 (Table S5 NDP by needs in the published supplementary information of the respective study). We narrowed livelihoods to be that generated from forest restoration by calculating the number of people employed as agricultural labourers and remanent workers not involved in primary agricultural and industrial employment sectors (data from (16)). We acknowledge that we do not account for the contentious issue of lack of accessibility of timber and non-timber-based forest products for basic needs, considering insecure land tenure and land rights of different peoples and communities in developing countries. To account for varying reliance of people living in rural and urban areas, we applied a simple continuous weighting (0-1) using information from Weiss et al., 2018., such that reliance increases as the time taken to reach the closest urban city increases (Fig S5; Equation 2). Additionally, we assumed that (i) houses constructed partly or entirely with wood are more durable compared to houses built using other natural materials like thatch, mud, clay (ii) fuel wood has the highest calorific value per unit of consumption for cooking purposes relative to charcoal, straw, animal dung etc.

$$people\ NCP = percentage\ societal\ reliance\ (\%) * population\ density (people km^{-2}) * time\ taken\ to\ reach\ nearest\ urban\ city\ centre\ (unitless)\ \ (normalized\ 0-1) \quad \text{(Equation 2)}$$

Note that, when required, all spatial data were resampled using bilinear interpolation method to 10km x10 km spatial resolution. All analyses were completed using R statistical software (18), unless otherwise specified.

(d) Distributional equity analyses

We first calculated the proportion of the total Indian population that identify as women and that belong to Scheduled Castes and Tribes in 5881 Indian subjuridictions for which socioeconomic data is available (16). We applied the proportions (women and socioeconomically disadvantaged people) to the total population in the restoration areas prioritized by all four plans.

Supplementary Tables

Table S1 Information to calculate biodiversity value NCP of the final set of 56 forest dependent mammals. Potential area of habitat (AOH) was calculated based on elevational limits and preferences of potential forest habitat type as per Brooks et al., 2016. We modified the target setting protocol from Jung et al., 2022 and Fastre et al 2021 (Tabe 1) to determine the preliminary target using information about the potential AOH on and around the restoration area in the range of each mammal. From the preliminary target we deducted the potential AOH outside the restoration area to calculate the final target. Percent target was calculated as the ratio of the final target and the potential AOH inside the restoration area. All AOH and target information is in sqkm and the Red List classification follows that of IUCN, 2020

Potential AOH	Potential AOH outside restoration area	Potential AOH inside restoration area	Total restoration area in range	Preliminary target	Final target	Mammal name	Red List classification	Percent target
2610.533	2190.842	420	473.0299	2200	9.15829	*Miniopterus pusillus*	LC	0.021805
16462.85	12824.62	3638.227	5608.267	13170.28	345.6592	*Macaca silenus*	EN	0.095008
12216.4	9493.608	2722.791	4325.853	9773.122	279.5145	*Platacanthomys lasiurus*	VU	0.102657
27353.14	21138.23	6214.909	11706.87	21882.51	744.2814	*Semnopithecus hypoleucos*	LC	0.119757
12732.59	9753.729	2978.864	3138.464	10186.07	432.3427	*Hypsugo affinis*	LC	0.145137
3748.598	2850.935	897.2727	962.9755	2998.879	147.9439	*Caracal caracal*	LC	0.164882
37067.43	27502.89	9564.545	14627.93	29653.95	2151.058	*Rhinolophus beddomei*	LC	0.224899
14939.18	10533.49	4405.7	5090.097	11951.34	1417.859	*Hipposideros pomona*	EN	0.321824
12708.18	5994.446	6713.736	13268.67	10166.55	4172.101	*Myotis peytoni*	DD	0.621428
10601.96	4742.543	5859.418	6564.653	8481.57	3739.027	*Viverra civettina*	CR	0.638123
168.809	152.3924	16.45455	16.41663	168.809	16.41663	*Myotis hasseltii*	LC	0.997695
1391.347	1380.097	11.27273	11.29039	1391.347	11.24972	*Cricetulus migratorius*	LC	0.997959
1017.018	1006.493	10.54545	5938.568	1017.018	10.52427	*Micromys minutus*	LC	0.997992
896.1173	663.8033	232.7273	276.7403	896.1173	232.3139	*Rhinolophus trifoliatus*	NT	0.998224
1073.857	1062.601	11.27273	12.34117	1073.857	11.25658	*Sicista concolor*	LC	0.998567
1797.304	1796.07	1.23636	14.81511	1797.304	1.23471	*Episoriculus leucops*	LC	0.998665
338.229	302.8002	35.45455	517.1508	338.229	35.42879	*Sorex minutus*	LC	0.999273
654.906	649.7638	5.14545	304.7258	654.906	5.14222	*Ochotona thibetana*	LC	0.999372
2100.645	2095.275	5.37273	76.29737	2100.645	5.37014	*Otomops wroughtoni*	DD	0.999518
1884.423	1856.254	28.18182	171.6578	1884.423	28.16837	*Apodemus rusiges*	LC	0.999523
1244.168	1223.086	21.09091	119.8626	1244.168	21.08254	*Alticola montosa*	VU	0.999603
249.7883	241.4455	8.34545	31.29618	249.7883	8.34276	*Mus famulus*	EN	0.999678
83.70929	83.40393	0.305455	895.109	83.70929	0.30536	*Rhinolophus subbadius*	LC	0.999689
386.7461	296.6823	90.09091	500.9803	386.7461	90.06382	*Rattus satarae*	VU	0.999699

1677.831	1672.268	5.56364	96.57099	1677.831	5.56224	*Niviventer brahma*	LC	0.999748
709.9824	703.1296	6.85455	528.0774	709.9824	6.85284	*Euroscaptor micrura*	LC	0.999751
47.21435	42.09704	5.11818	297.7262	47.21435	5.11731	*Chimarrogale himalayica*	LC	0.99983
143.6597	141.3207	2.33636	1186.235	143.6597	2.33906	*Ailurus fulgens*	EN	1
59.85035	56.21315	3.63636	48.49475	59.85035	3.6372	*Alticola stoliczkanus*	LC	1
241.6425	239.9697	1.67273	1.67288	241.6425	1.67287	*Capra falconeri*	NT	1
132.3157	123.0147	9.27273	581.3877	132.3157	9.30094	*Dacnomys millardi*	DD	1
1673.276	1671.039	2.23636	14.04306	1673.276	2.23668	*Eothenomys melanogaster*	LC	1
511.3766	494.6333	16.72727	30.24841	511.3766	16.74325	*Hadromys humei*	EN	1
2174.908	2145.32	29.54545	203.18	2174.908	29.58803	*Hyperacrius fertilis*	NT	1
1789.608	1760.145	29.45455	340.2448	1789.608	29.46274	*Hyperacrius wynnei*	LC	1
2170.774	1996.014	174.5455	1483.114	2170.774	174.7594	*Latidens salimalii*	EN	1
974.5008	972.4238	2.07273	10.68154	974.5008	2.07697	*Leopoldamys sabanus*	LC	1
1448.449	1411.17	37.27273	60.50815	1448.449	37.27918	*Phaiomys leucurus*	LC	1
1643.795	1633.7	10.09091	395.3791	1643.795	10.09484	*Neodon sikimensis*	LC	1
1353.163	1349.498	3.66364	144.4196	1353.163	3.66528	*Murina aurata*	DD	1
3.52297	3.51505	0.007918	871.4273	3.52297	0.00792	*Myotis sicarius*	VU	1
291.5217	143.9414	147.2727	1093.889	291.5217	147.5803	*Nesokia indica*	LC	1
158.3695	154.151	4.21818	764.5122	158.3695	4.21851	*Nyctalus montanus*	LC	1
4.46593	4.25846	0.207273	0.207	4.46593	0.20747	*Panthera leo*	VU	1
725.3597	624.3631	100.9091	228.9117	725.3597	100.9966	*Suncus montanus*	VU	1
1341.819	1321.987	19.81818	31.23734	1341.819	19.83131	*Trachypithecus geei*	EN	1
74.83304	58.07149	16.72727	230.9818	74.83304	16.76155	*Episoriculus macrurus*	LC	1
219.8541	219.1301	0.723636	5.52728	219.8541	0.72402	*Anourosorex squamipes*	LC	1
1966.094	1931.989	34.09091	1575.962	1966.094	34.10514	*Arielulus circumdatus*	LC	1
32.38139	32.34411	0.037273	37391.46	32.38139	0.03728	*Sus scrofa*	LC	1
298.2563	247.699	50.54545	404.1168	298.2563	50.55732	*Vandeleuria nilagirica*	EN	1
800.1285	743.4618	56.63636	92.24721	800.1285	56.66663	*Macaca munzala*	EN	1
277.5441	275.213	2.32727	73.59079	277.5441	2.33107	*Anourosorex schmidi*	DD	1
1753.81	1749.771	4.03636	321.2197	1753.81	4.03875	*Anourosorex assamensis*	LC	1
173.0565	171.7824	1.27273	61.82149	173.0565	1.2741	*Petaurista mishmiensis*	NT	1
22.15497	21.4237	0.730909	348.5442	22.15497	0.73127	*Murina tubinaris*	DD	1

Table S2 Description of datasets used in this study

Dataset Reference	Brief description	Analytical step	Purpose in this study
(2) Locational information of different types of natural forests across India	Seamless vegetation map of India with 100 vegetation classes. This map was made using ground control points of presences of all vegetation types in India. The resulting seamless vegetation map is publicly available at- - https://zenodo.org/doi/10.5281/zenodo.11615010	Modelling biophysical envelope of forests	We used the 15565 ground control points (under a data-sharing agreement as per Biodiversity Act of India 2002) filtered for presence of natural forests only (final n=10756) to develop the biophysical envelope of forest cover.
(19) ECMWF Reanalysis v5 (ERA5) (freely available and accessible through Google Earth Engine)	Global climate data covering periods Jan 1940 to present (spatial resolution=0.1 deg). We used precipitation and temperature data for 1981-2010.	Modelling biophysical envelope of forests	We calculated mean annual precipitation, mean annual temperature, temperature seasonality, precipitation seasonality as per (20). Additionally we calculated maximum climate water deficit, a nuanced measure of severity of water stress which is an ecologically relevant metric as per (21).
(22) ISRIC World SoilGrids (0-30cm depth) (data published and freely available)	Global database of soil properties. We extracted percentage of silt, clay, sand and coarse fragments	Modelling biophysical envelope of forests	We estimated percentage of the considered soil properties considering the weighted sum across all soil profiles (by including bulk density information provided in the database)
(23) Elevation information (freely available and accessible through Google Earth Engine)	Global digital surface model from which we extracted elevation and slope information	Modelling biophysical envelope of forests	Elevation and topography were calculated from the digital elevation models using Google Earth Engine.
(5) Copernicus Global Land Cover CGLS-LC100 Collection 3 (freely available)	Global dynamic land cover map at 100 m spatial resolution with overall accuracy of 80%.	Sequential exclusion of LULCs that cannot be restored to forests	Used to exclude unknown, urban/built-up, bare/sparse vegetation, snow & ice, permanent water bodies, herbaceous wetlands, moss & lichen, oceans & seas and cultivated and managed vegetation/ agriculture.
(6) (freely available)	India specific decadal land use and land cover classification across India for 1985, 1995 and 2005 (only 2005 used in our study)	Sequential exclusion of LULCs that cannot be restored to forests	Used to exclude all deciduous & evergreen broadleaf forests, deciduous & evergreen needle leaf forests, mixed forests and grasslands

(7) (data published and freely available)	Global study about the carbon sequestration rates (above+ belowground) of native forests for 30 years of growth	Climate NCP	Used the India specific carbon sequestration rates.
(8) (freely available)	Global inventory of the global conservation status and extinction risk of biological species	Biodiversity NCP	Used species range polygons for all mammals who habitat preference is forest within the Indian jurisdiction. We also extracted elevational limits and preferred forest habitat type information to determine potential area of habitat.
(15) (data published and freely available)	Societal reliance information for basic needs of housing construction material, energy, livelihoods and water resources	Societal NCP	Used percentage reliance for three needs- housing construction material, energy and livelihoods for each Indian level 2 sub jurisdiction.
(17) (data published and freely available)	Global map of travel time to cities (2015)	Societal NCP	Used to weight the societal reliance to differentiate reliance on forests between people living in rural and urban areas.
(16) SHRUG database (data published and freely available)	Repository of socioeconomic information at the Indian village level (finest spatial scale of social data collection)	Distributional equity and societal NCP	Used the 2011 Population Census Abstract information as part of the Population census data. For each Indian level 3 sub jurisdiction, we extracted the total population (pc11_pca_tot_p), total SC population (pc11_pca_p_st), total SC population (pc11_pca_p_sc) and the total female population (pc11_pca_tot_f).<br><br>Furthermore, to determine livelihoods from non-agriculture and non-industry sectors, we extracted information about marginal and main workers agricultural labourers and other workers (pc11_pca_main_al_m, pc11_pca_main_al_f, pc11_pca_marg_al_p, pc11_pca_main_ot_p, pc11_pca_marg_ot_p).

Supplementary Figures

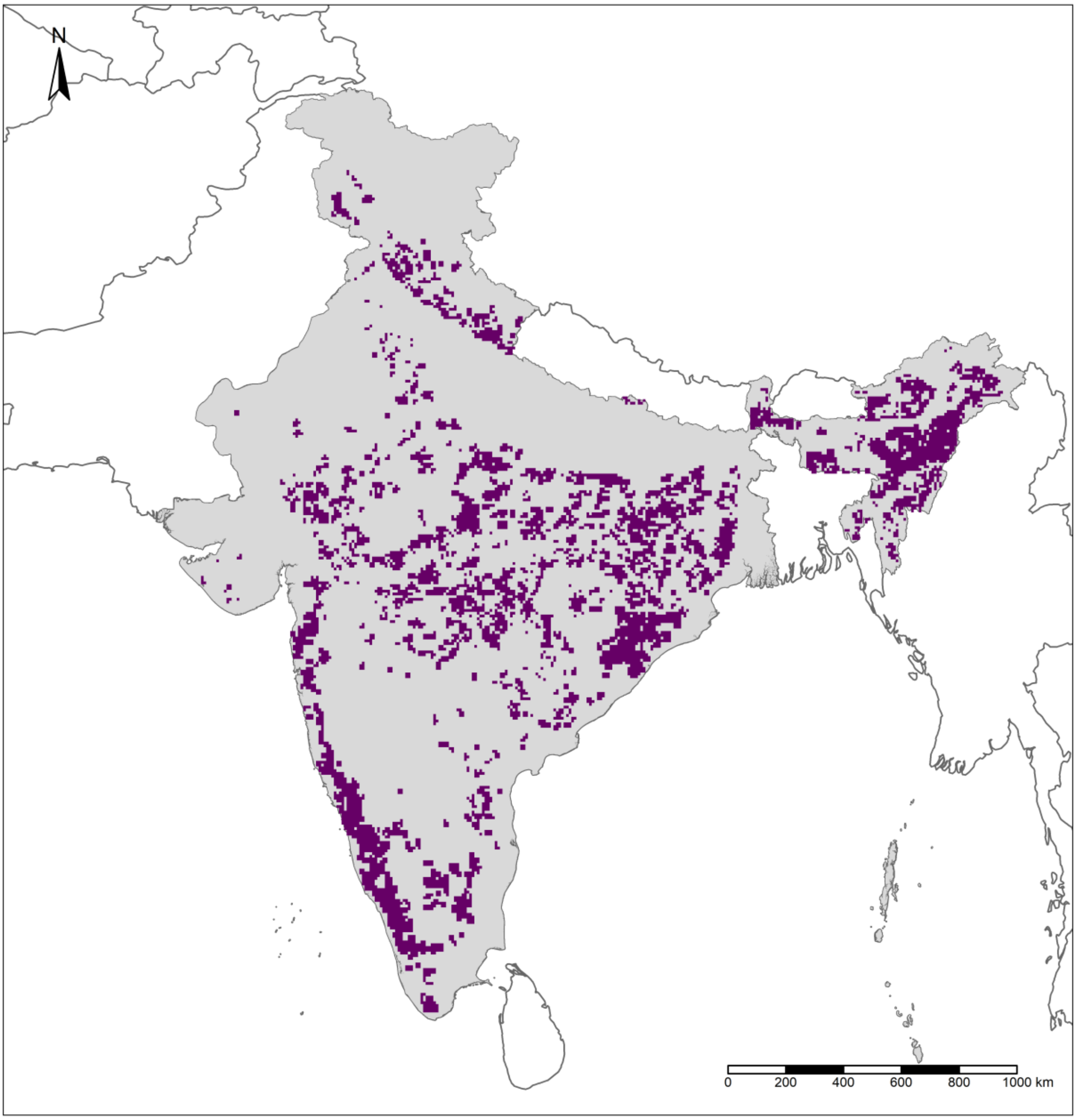

Figure S1 3.88 Mha restoration area considered in the study (purple) from an initial study area shown in grey

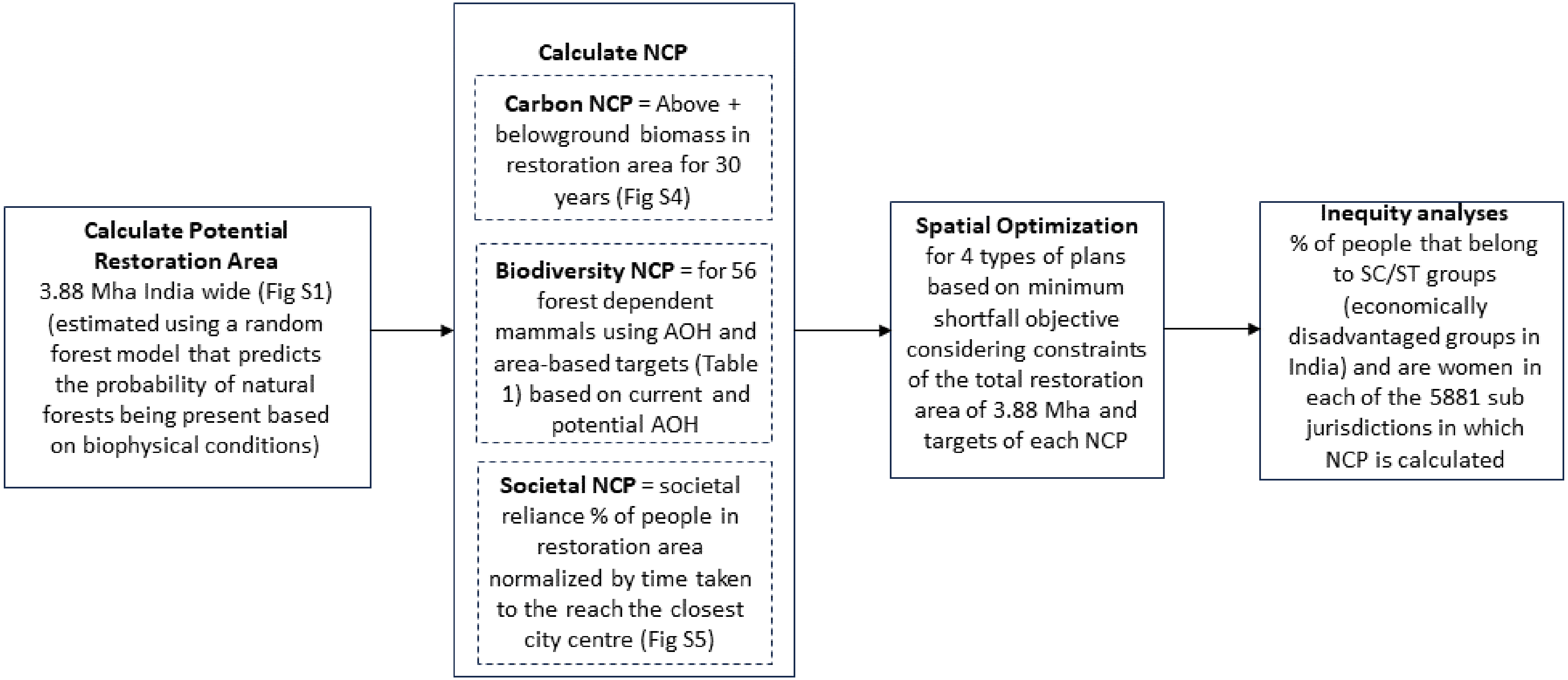

Figure S2 Brief schematic of workflow including the estimation of restoration area of 3.88 Mha (Figure S1), calculation of the different NCP in the restoration area, spatial optimization considering the 4 types of plans and assessment of inequality considering socio-economically disadvantaged Indians and women

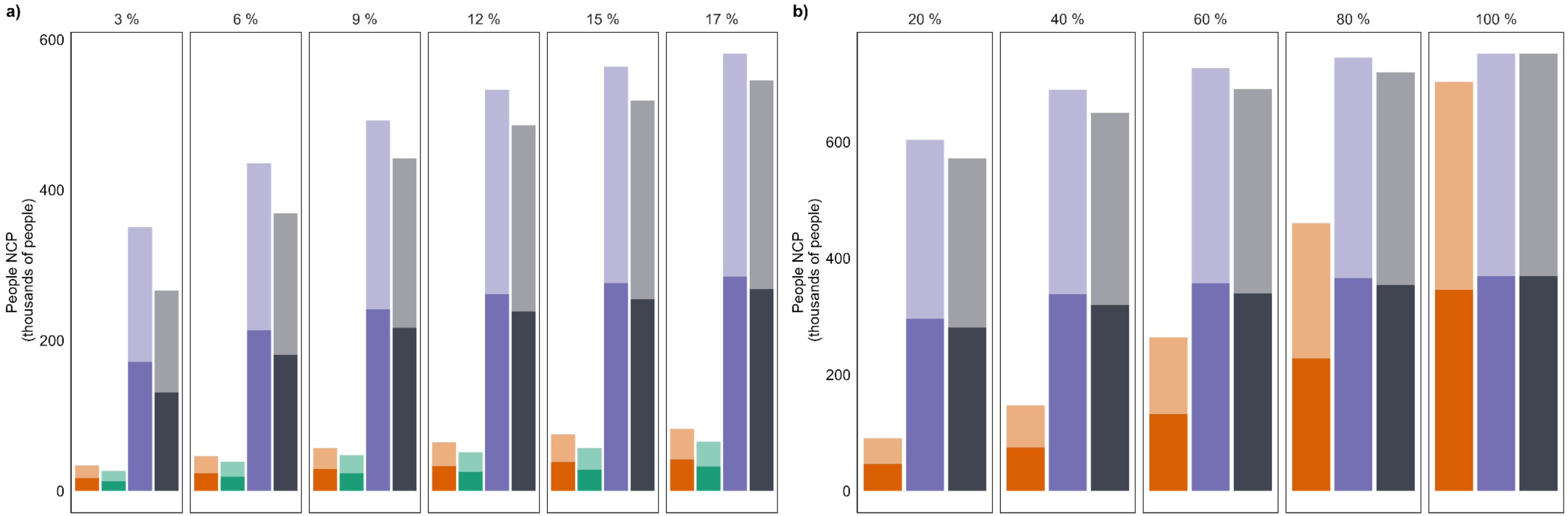

Figure S3 Distributional equity of women following similar interpretation as Fig 3

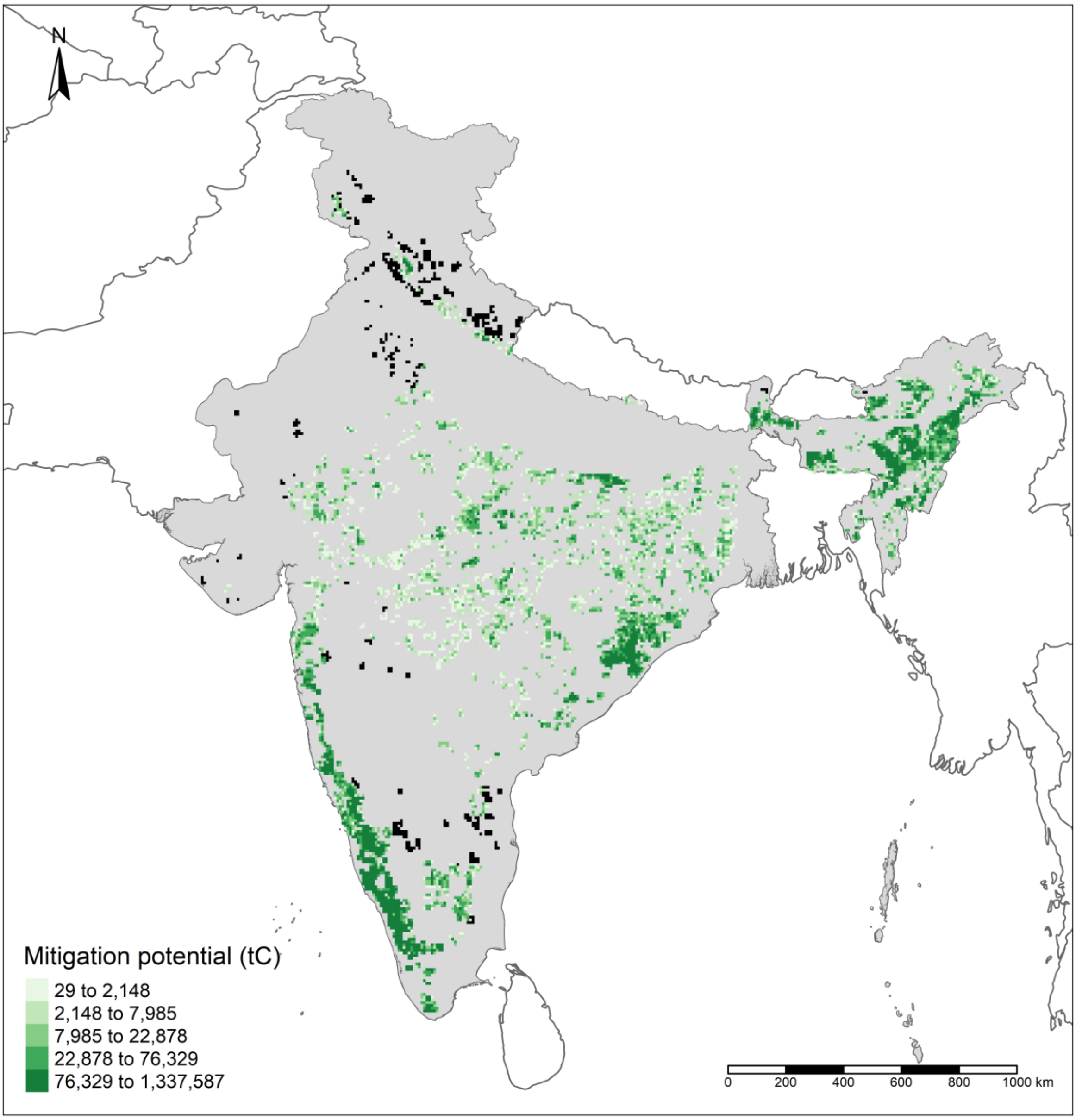

Figure S4 Climate NCP calculated in the restoration area (Fig S1). 4.6% of the total restoration area (black) does not have information about aboveground and/or belowground carbon sequestration from Cook-Patton et al., 2020. These areas were included in the analyses assuming that these areas would deliver biodiversity value and societal NCP.

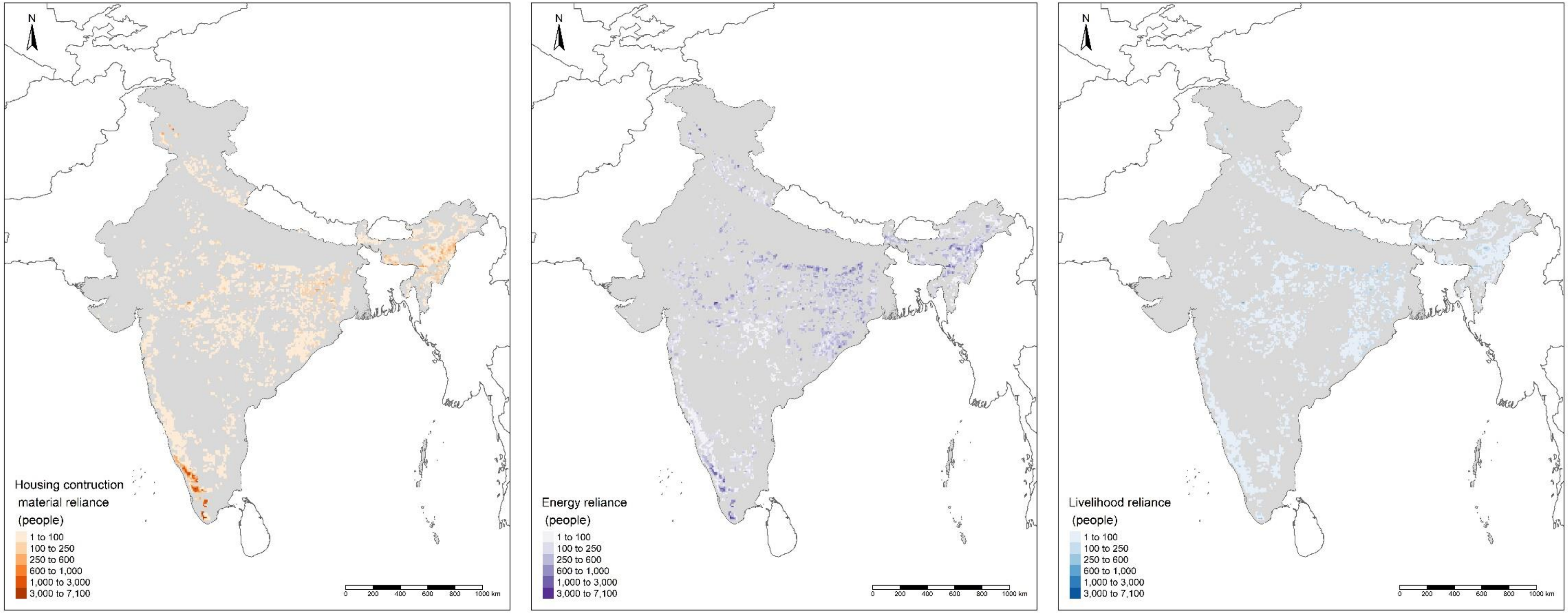

Figure S5 Societal NCP determined in the restoration area (Fig S1)

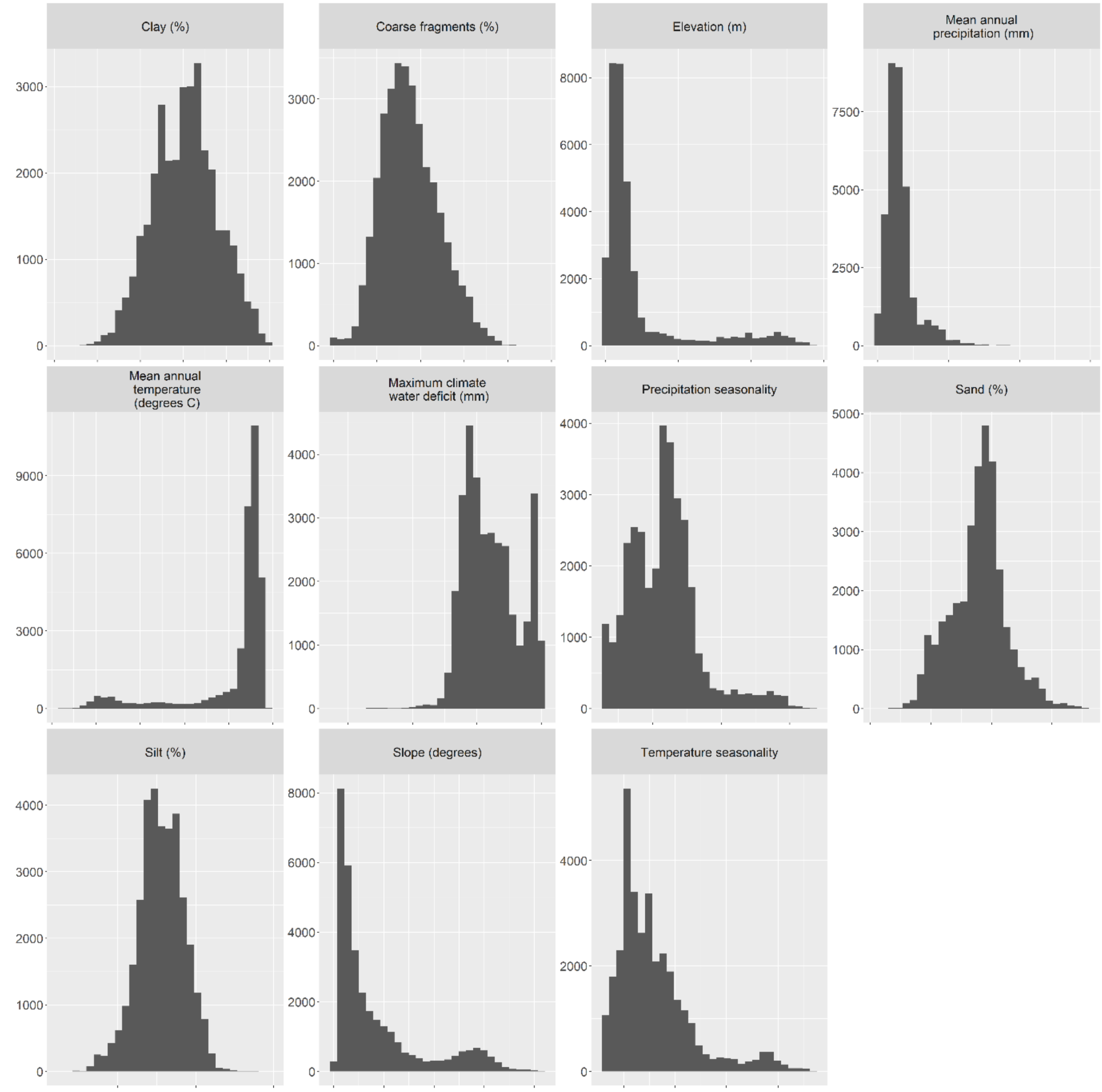

Figure S6 Distribution of environmental variables considered across the 10756 presences and 22453 pseudoabsences, used to map the biophysical envelope of native forests across India as per Gopalakrishna et al., 2022

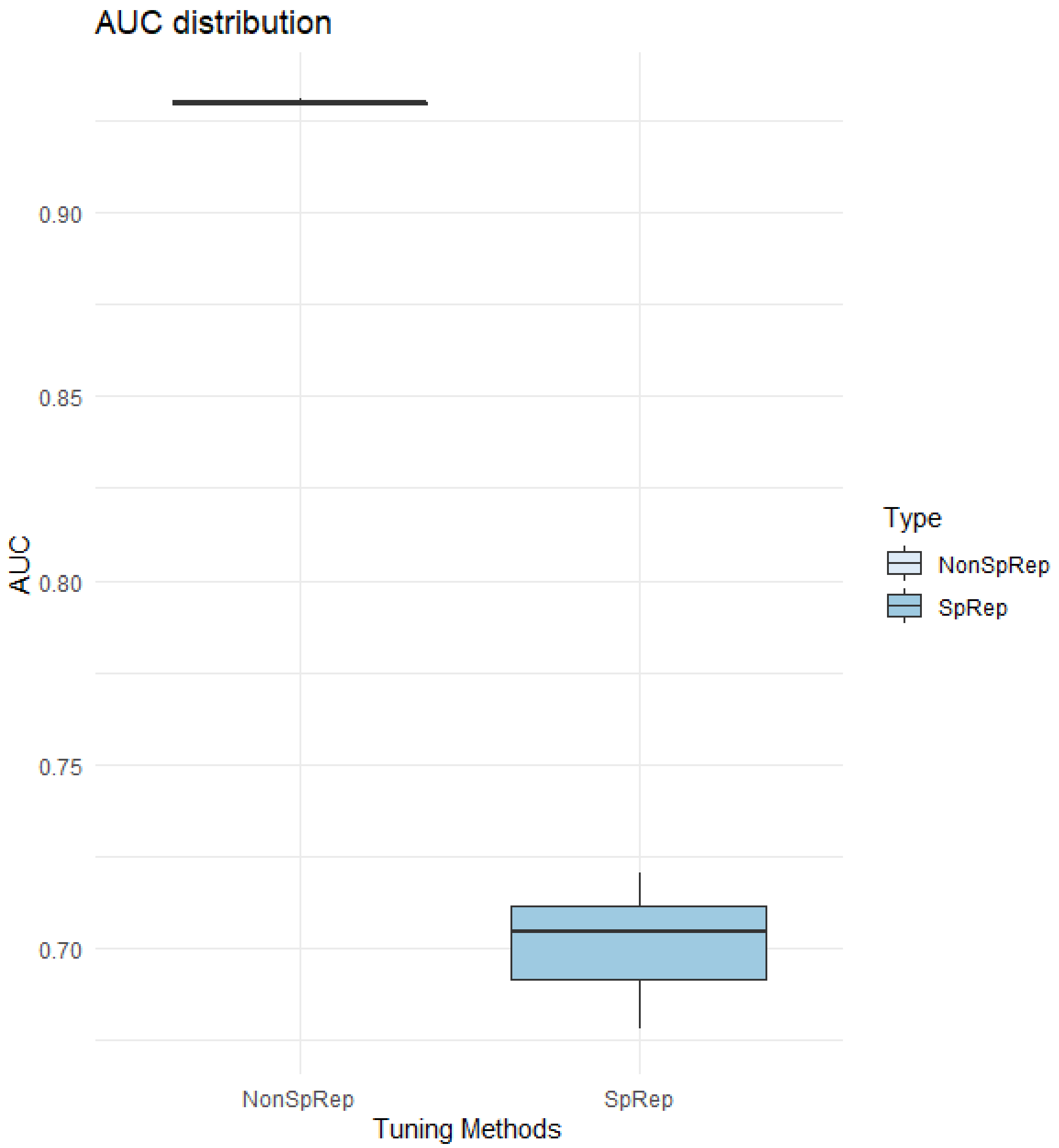

Figure S7 Parameterization of random forest algorithm using spatial repeated cross validation (SpRep) with reduced AUC=0.702 when compared to non-spatial repeated cross validation (NonSpRep)

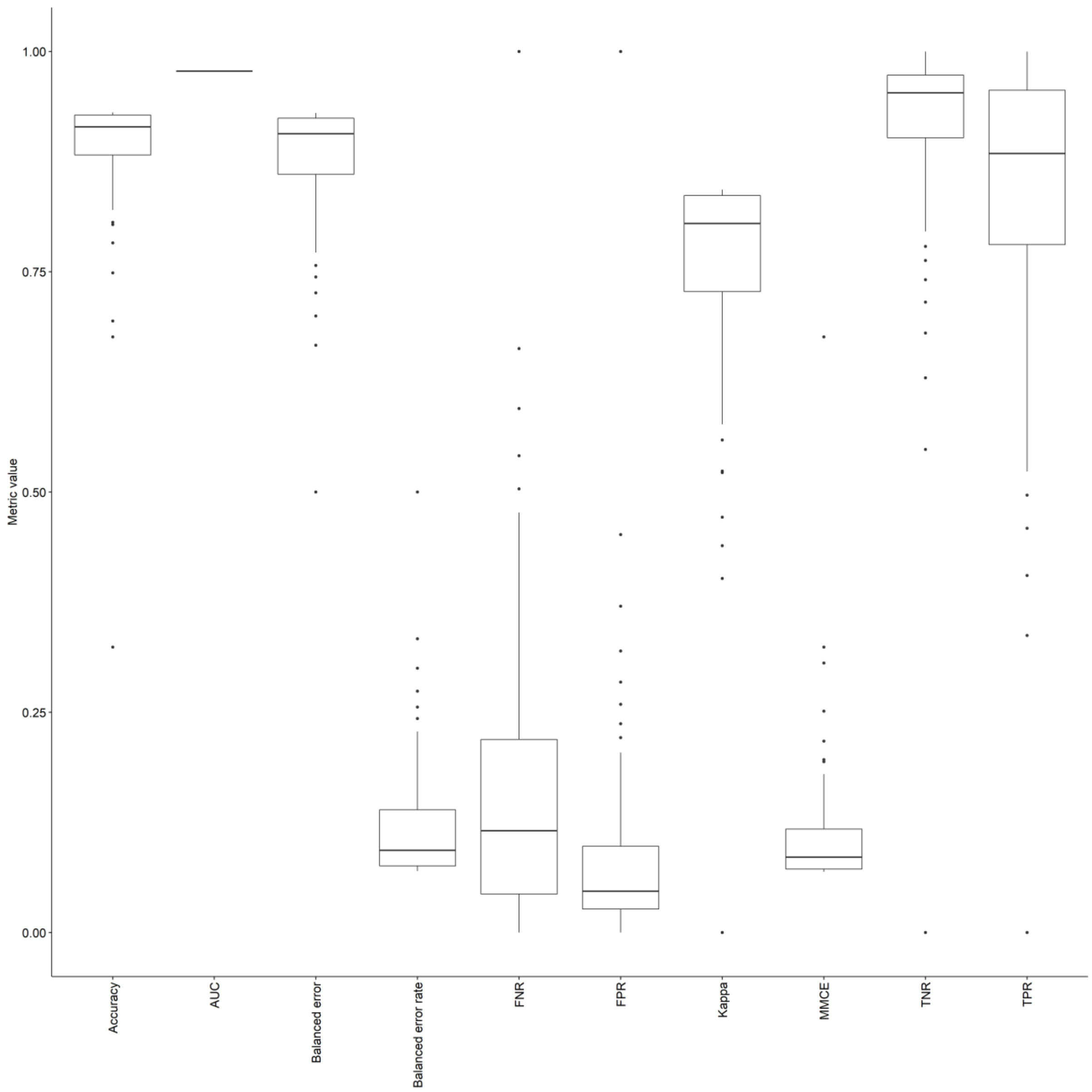

Figure S8 Distribution of the 10 accuracy metrics across all runs. The presence threshold=0.494 was used as it corresponds to the least mean misclassification error (MMCW)=0.069

References

1. T. Gopalakrishna, *et al.*, Existing land uses constrain climate change mitigation potential of forest restoration in India. *Conserv. Lett.*, 1–11 (2022).

2. P. S. Roy, *et al.*, New vegetation type map of India prepared using satellite remote sensing: Comparison with global vegetation maps and utilities. *Int. J. Appl. Earth Obs. Geoinf.* **39**, 142–159 (2015).

3. M. Barbet-Massin, F. Jiguet, C. H. Albert, W. Thuiller, Selecting pseudo-absences for species distribution models: How, where and how many? *Methods Ecol. Evol.* **3**, 327–338 (2012).

4. P. Schratz, J. Muenchow, E. Iturritxa, J. Richter, A. Brenning, Performance evaluation and hyperparameter tuning of statistical and machine-learning models using spatial data (2018) https:/doi.org/10.1016/j.ecolmodel.2019.06.002.

5. M. Buchhorn, *et al.*, Copernicus Global Land Cover Layers—Collection 2. *Remote Sens.* **12**, 1044 (2020).

6. P. S. Roy, *et al.*, Development of decadal (1985--1995--2005) land use and land cover database for India. *Remote Sens.* **7**, 2401–2430 (2015).

7. S. C. Cook-Patton, *et al.*, Mapping carbon accumulation potential from global natural forest regrowth. *Nature* **585**, 545–550 (2020).

8. IUCN, "The IUCN Red List of Threatened Species" (2020).

9. M. Jung, *et al.*, A global map of terrestrial habitat types. *Sci. Data* **7**, 1–8 (2020).

10. R. Crouzeilles, *et al.*, Achieving cost-effective landscape-scale forest restoration through targeted natural regeneration. *Conserv. Lett.* (2020) https:/doi.org/10.1111/conl.12709 (March 9, 2020).

11. T. M. Brooks, *et al.*, Measuring Terrestrial Area of Habitat (AOH) and Its Utility for the IUCN Red List. *Trends Ecol. Evol.* **34**, 977–986 (2019).

12. C. Fastré, W. J. van Zeist, J. E. M. Watson, P. Visconti, Integrated spatial planning for biodiversity conservation and food production. *One Earth* **4**, 1635–1644 (2021).

13. M. Jung, *et al.*, Areas of global importance for conserving terrestrial biodiversity, carbon and water. *Nat. Ecol. Evol.* (2021) https:/doi.org/10.1038/s41559-021-01528-7.

14. Center for International Earth Science Information Network - CIESIN - Columbia Univeristy, Gridded Population of the World, Version 4 (GPWv4): Population Density, Revision 11 (2018).

15. G. Fedele, C. I. Donatti, I. Bornacelly, D. G. Hole, Nature-dependent people: Mapping human direct use of nature for basic needs across the tropics. *Glob. Environ. Chang.* **71**, 102368 (2021).

16. S. Asher, T. Lunt, R. Matsuura, P. Novosad, Development Research at High Geographic Resolution: An Analysis of Night Lights, Firms, and Poverty in India using the SHRUG Open Data Platform. *World Bank Econ. Rev.* (2021).

17. D. J. Weiss, *et al.*, A global map of travel time to cities to assess inequalities in accessibility in 2015. *Nature* **553**, 333–336 (2018).

18. R Core Team, R: A Language and Environment for Statistical Computing (2020).

19. Copernicus Climate Change Service Climate Data Store (CDS), ERA5: Fifth generation of

ECMWF atmospheric reanalyses of the global climate (2017) (January 5, 2020).

20. M. S. O'donnell, D. A. Ignizio, "Bioclimatic Predictors for Supporting Ecological Applications in the Conterminous United States Data Series 691."

21. L. E. O. C. Aragão, *et al.*, Spatial patterns and fire response of recent Amazonian droughts. *Geophys. Res. Lett.* **34**, 1–5 (2007).

22. T. Hengl, *et al.*, SoilGrids250m: Global gridded soil information based on machine learning. *PLoS One* **12** (2017).

23. E. Jarvis, A., Reuter, H.I., Nelson, A. and Guevara, Hole-filled SRTM for the globe Version 4. 25–54 (2008).
